# Does Point-of-care Ultrasonography Change Emergency Department Care Delivered to Hypotensive Patients When Categorized by Shock Type? A Post-Hoc Analysis of an International Randomized Controlled Trial from the SHoC-ED Investigators

**DOI:** 10.7759/cureus.6058

**Published:** 2019-11-03

**Authors:** Paul Atkinson, Sam Hunter, Ankona Banerjee, David Lewis, Jacqueline Fraser, James Milne, Laura Diegelmann, Hein Lamprecht, Melanie Stander, David Lussier, Chau Pham, Mandy Peach, Luke Taylor, Ryan Henneberry, Michael Howlett, Jay Mekwan, Brian Ramrattan, Joanna Middleton, Daniel J Van Hoving, George Stoica, James French, Paul Olszynski

**Affiliations:** 1 Emergency Medicine, Saint John Regional Hospital, Saint John, CAN; 2 Science, University of Ottawa, Ottawa, CAN; 3 Medical Services, WorkSafeNB, Saint John, CAN; 4 Emergency Medicine, Dalhousie University, Saint John, CAN; 5 Dalhousie University, Sydney, Nova Scotia, CAN; 6 Emergency Medicine, University of Maryland, Baltimore, USA; 7 Emergency Medicine, Stellenbosch University, Cape Town, ZAF; 8 Emergency Medicine, Mediclinic, Cape Town, ZAF; 9 Emergency Medicine, University of Manitoba, Winnipeg, CAN; 10 Emergency Medicine, University of Manitoba, Winnipeg, CAN; 11 Emergency Medicine, Saint John, CAN; 12 Emergency Medicine, Dalhousie University, Halifax, CAN; 13 Emergency Medicine, Horizon Health Network, Saint John, CAN; 14 Emergency Medicine, Saint John Regional Hospital/, Saint John, CAN; 15 Research Services, Horizon Health Network, Saint John, CAN; 16 Emergency Medicine, University of Saskatchewan, Saskatoon, CAN

**Keywords:** point-of-care ultrasound, hypotension, shock, interventions

## Abstract

Introduction

Our previously reported randomized-controlled-trial of point-of-care ultrasound (PoCUS) for patients with undifferentiated hypotension in the emergency department (ED) showed no survival benefit with PoCUS. Here, we examine the data to see if PoCUS led to changes in the care delivered to patients with cardiogenic and non-cardiogenic shock.

Methods

A post-hoc analysis was completed on a database of 273 hypotensive ED patients randomized to standard care or PoCUS in six centres in Canada and South Africa. Shock categories recorded one hour after the ED presentation were used to define subcategories of shock. We analyzed initial intravenous fluid volumes, as well as rates of inotrope use and procedures.

Results

261 patients could be classified as cardiogenic or non-cardiogenic shock types. Although there were expected differences in the mean fluid volume administered between patients with non-cardiogenic and cardiogenic shock (p-value<0.001), there was no difference between the control and PoCUS groups (mean non-cardiogenic control 1881mL (95% CI 1567-2195mL) vs non-cardiogenic PoCUS 1763mL (1525-2001mL); and cardiogenic control 680mL (28.4-1332mL) vs. cardiogenic PoCUS 744mL (370-1117mL; p= 0.67). Likewise, there were no differences in rates of inotrope administration nor procedures for any of the subcategories of shock between the control group and PoCUS group patients.

Conclusion

Despite differences in care delivered by subcategory of shock, we did not find any difference in key elements of emergency department care delivered between patients receiving PoCUS and those who did not. This may help explain the previously reported lack of outcome differences between groups.

## Introduction

Patients who present to the emergency department (ED) with non-traumatic hypotension and shock have high mortality rates and pose both diagnostic and therapeutic challenges for emergency physicians [1-2]. Early appropriate management may provide clinical outcome benefits [3-4]. Although the use of point of care ultrasound (PoCUS) protocols for patients with undifferentiated hypotension in the ED is widespread [5], our previously reported international randomized controlled trial (RCT), the Sonography in Hypotension and Cardiac Arrest in the Emergency Department (SHoC-ED) study, showed no clear survival or length-of-stay benefit for patients assessed with PoCUS [6]. There is some evidence that the use of PoCUS in shock can lead to altered treatment plans [2]. In this analysis of the SHoC-ED study [6], we examine how the randomization to the PoCUS or control group affected actual care delivered to patients with each major category of shock. We examine the impact on fluid bolus administration in addition to other emergency interventions. The primary comparison was care delivered during resuscitation between patients who had a working diagnosis of cardiogenic versus non-cardiogenic shock types. We wondered if the absence of outcome benefits previously reported might stem from a lack of impact of PoCUS on the actual care delivered between patients with different types of shock.

## Materials and methods

A planned post hoc analysis was conducted on a database of 273 patients with undifferentiated hypotension who had completed follow-up in the previously reported SHoC-ED study [6]. This was an international multicentre randomized controlled trial that recruited at six emergency care sites: three in Canada, and three in South Africa. Subjects were adult patients with undifferentiated hypotension (SBP<100 or shock index>1), randomized to early PoCUS plus standard care versus standard care without PoCUS. Diagnoses were recorded at 0 and 60 minutes as well as by a blinded chart review at hospital discharge. The primary outcome measure was survival to 30 days or hospital discharge. Secondary outcome measures included initial treatment including the key interventions of initial intravenous (IV) fluid volume, frequency of inotrope administration, and frequency of recorded procedures, as well as investigations, admissions, and length of stay. Patients were analyzed based on their randomized groups; those who received PoCUS and those in the control group who did not receive PoCUS. The patients were also grouped by the initial working impression of their type of shock as recorded at the repeat assessment mark, 60 minutes post-ED arrival. At this point, they would have received either an initial and repeat clinical assessment (control group), or similar plus completion of a standardized PoCUS protocol (PoCUS group). The recorded shock type at this point would best indicate the treating physician's impression and rationale for the treatments provided during ED resuscitation. Patients were grouped by their main category of shock; either cardiogenic or non-cardiogenic. The data was analyzed to determine if care delivered varied by major shock category, dependent upon the use of PoCUS. The study was registered at ClinicalTrials.gov (registration number NCT01419106) and all sites received local research ethics board (REB) approval. The work has been previously presented at the Canadian Association of Emergency Physicians' national scientific conference, 2019, and the abstract published in the proceedings of that meeting [7].

Participants

Included subjects were adult patients, 19 and older, who were deemed to be in a shocked state based on either of two parameters: a presentation with a sustained initial systolic blood pressure <100, or a shock index >1.0 (with a systolic blood pressure < 120 mmHg). Shock index (SI) is defined as heart rate over systolic blood pressure (SBP). Exclusion criteria were: pregnancy at time of presentation or discovered during initial screening; CPR or advanced cardiac life support interventions (e.g. defibrillation, emergency pacing, insertion of ventricular assist device, etc.) prior to screening or enrolment; a history of significant trauma in past 24 hours; a 12-lead electrocardiogram (ECG) diagnostic of acute myocardial infarction (AMI); a clear mechanism or etiology for the hypotension or shock is evident (i.e. the patient does not have undifferentiated shock), a previously known diagnosis from another hospital (for transferred patients); a vagal episode (as cause of hypotension) and low blood pressure considered to be non-pathologic (normal variant or other).

Materials

The original SHoC-ED study database information on actual care delivered [6] was analyzed using R software [R Core Team (2017), R Foundation for Statistical Computing, Vienna, Austria (https://www.R-project.org/)].

## Results

**Figure 1:**
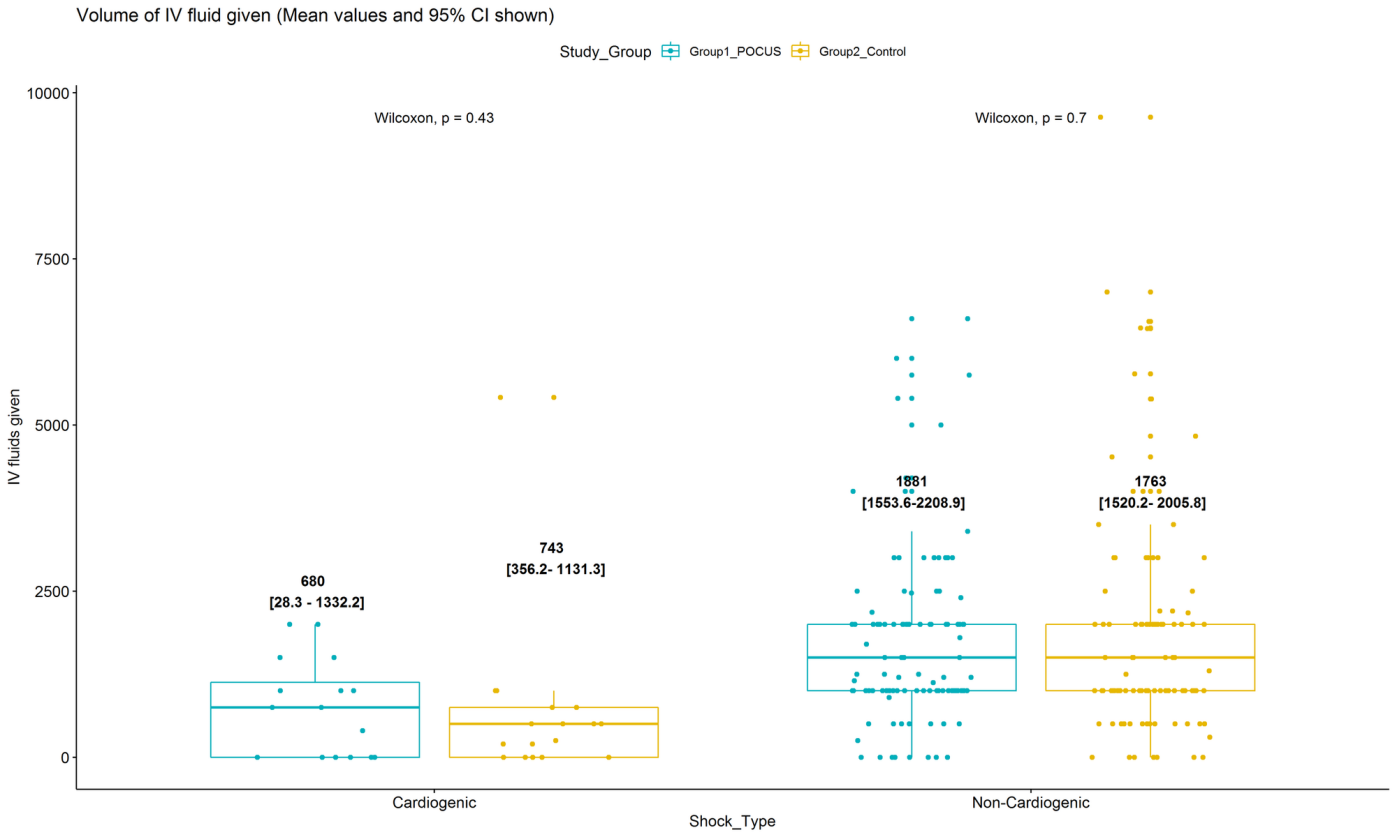
Mean values of recorded volumes of intravenous (IV) fluids administered in the emergency department PoCUS: point-of-care ultrasound; CI: confidence intervals; n: number; IV: intravenous

Baseline characteristics were similar in the PoCUS and control groups, as reported previously [6] and shown in Table 1. Although patients with cardiogenic shock received smaller mean volumes of intravenous fluid during their ED resuscitation than patients with non-cardiogenic shock, there was no difference within these shock categories between patients who had been randomized to the PoCUS and control groups, as detailed in Table 1 and Figure 1.

**Table 1:** Baseline demographic profile of study participants and primary outcome of initial mean fluid bolus volumes administered to patients diagnosed with cardiogenic and non-cardiogenic shock PoCUS: point-of-care ultrasound; CI: confidence intervals; n: number; IQR: inter-quartile range; ED: emergency department; HR: heart rate; SBP: systolic blood pressure

Variable	PoCUS	Control
Total Participants (n)	138	135
North America n (%; 95% CI)	90 (65.2%; 56.6 to 73.1%)	89 (65.9%; 57.2 to 73.8%)
South Africa n (%; 95% CI)	48 (34.8%; 26.8 to 43.3%)	46 (34.1%; 26.1 to 42.7%)
Male n (%; 95% CI)	73 (52.9%; 44.2 to 61.4%)	65 (48.1%; 39.4 to 56.9%)
Age in years: Median (IQR)	56 (53.4 to 59.8)	58.5 (56.2 to 62.1)
SBP in mmHg: Median (IQR)	91.0 (88.5 to 94.2)	91.8 (89.1 to 94.8)
HR in bpm: Median (IQR)	106.5 (102.4 to 111.8)	111.4 (105.8 to 116.5)
Resps in bpm: Median (IQR)	24.3 (22.3 to 26.0)	23.9 (22.8 to 25.6)
Temp in deg C: Median (IQR)	36.7 (36.5 to 36.9)	36.8 (36.6 to 37.0)
Mean Volume ED Fluid Bolus Recorded in Cardiogenic shock patients (n=33); mL (95% CI)	744 (356 to 1131)	680 (28 to 1332)
Mean Volume ED Fluid Bolus Recorded in Non-Cardiogenic shock patients (n=214); mL (95% CI)	1763 (1520 – 2006)	1881 (1554 to 2209)

Cardiogenic PoCUS group patients received 744 mL (95% confidence interval [CI] 356 to 1131), similar to the 680 mL (28.4 to 1332.2) for cardiogenic control group patients. Both non-cardiogenic groups received more than cardiogenic groups with 1763 mL (1520 - 2006) for non-cardiogenic PoCUS; and 1881 mL (1554 to 2209) for non-cardiogenic control group patients. ANOVA comparisons between groups are shown in Table 2.

**Table 2:** Number of patients and rates undergoing major procedures, in PoCUS and control groups, including the number of patients in each category of shock PoCUS: point-of-care ultrasound; CI: confidence intervals; n: number

Type of Shock (n)	POCUS (n); %; 95% CI)	Control (n); %; 95% CI)
Cardiogenic (34) Fisher’s p-value= 0.69	5 (29.4; 7.7 to 51.1%)	3 (17.6; -0.5 to 35.8%)
Non-Cardiogenic (227) Chi Sq. <0.01; p= 0.97	20 (17.7; 10.7 to 24.7%)	20 (17.8; 10.6 to 24.5%)

For secondary outcomes, no meaningful difference was seen within shock categories for the frequency of inotrope administration for patients in either group: PoCUS-Cardiogenic 17.6% (-0.4 to 35.8%) vs Control-Cardiogenic 11.8% (-3.5 to 27.1%); and PoCUS-Non-cardiogenic 12.4% (6.3 to 18.5%) vs Control-Non-cardiogenic 8.8% (3.6 to 13.9%). There was no significant difference in the rates of ED procedures delivered between the groups: PoCUS-Cardiogenic 29.4% (7.7 to 51.1%) vs Control-Cardiogenic 17.6% (-0.5 to 35.8%); and PoCUS-Non-cardiogenic 17.7% (10.7 to 24.7%) vs Control-Non-cardiogenic 17.8% (10.6 to 24.5%). Absolute numbers for these results are shown in Table 3 and Table 4.

**Table 3:** ANOVA comparing differences in fluid bolus recorded between patients grouped by shock category and PoCUS or control PoCUS: point-of-care ultrasound; CI: confidence intervals; ANOVA: analysis of variance

Categories compared	Difference (ml; 95% CI)	P-value
Cardiogenic POCUS vs Cardiogenic Control	63 (-1240 to 1367)	0.99
Non-Cardiogenic POCUS vs Cardiogenic Control	1083 (107 to 2058)	0.02*
Non-Cardiogenic Control vs Cardiogenic Control	1201(223 to 2179)	0.01*
Non-Cardiogenic POCUS vs Cardiogenic POCUS	1019 (17 to 2021)	0.04*
Non-Cardiogenic Control vs Cardiogenic POCUS	1137 (133 to 2142)	0.02*
Non-Cardiogenic Control vs Non-Cardiogenic POCUS	118 (-393 to 630)	0.93

**Table 4:** Number of patients and rates receiving inotropes, in PoCUS and control groups, including the number of patients in each category of shock POCUS: point-of-care ultrasound; CI: confidence intervals; n: number

Type of Shock (n)	POCUS (n; %; 95% CI)	Control (n; %; 95% CI)
Cardiogenic (34) Fisher’s p= 0.99	3 (17.6; -0.4 to 35.8%)	2 (11.8; -3.5 to 27.1%)
Non- Cardiogenic (227) Chi-Sq. 0.78; p= 0.37	14 (12.4; 6.3 to 18.5%)	10 (8.8; 3.6 to 13.9%)

## Discussion

In hypotensive patients assessed with PoCUS during their ED resuscitation, there was a clear difference in the approach to fluid administration between patients with various categories of shock, with patients in cardiogenic shock receiving smaller volumes of intravenous fluid than non-cardiogenic shock patients. However, this pattern was also seen in the control group, who received ED assessment and usual care without PoCUS. It is apparent that despite PoCUS providing additional information relating to diagnosis, shock type, and volume status, clinicians were able to differentiate between shock types without PoCUS, providing appropriate volumes of fluid and rates of interventions utilizing clinical skills and other standard technologies available in the emergency department. While it is possible that the volumes of fluid actually provided could have been influenced by initial routine fluid administration by nursing protocols for critically ill hypotensive patients, this is unlikely to account for the lack of difference between PoCUS and control groups, especially with a clear difference in volumes administered for cardiogenic and non-cardiogenic shock types. 

The analysis showing that patients with each type of shock received similar care independent of allocation to the PoCUS or control group may help to explain why there was no difference in clinical outcomes such as survival and length of stay, as well as various other secondary outcomes in the previously published SHoC-ED study [6].

The ability to compare treatment and interventions with a control group of patients helped us to assess if the use of PoCUS, or other clinical assessment skills, were responsible for differences in treatment for patients in each shock category. Future research into the impact of point of care interventions may benefit from a prospective comparative study design. A brief clinician's capsule is provided in Appendix 1.

Limitations

When considering any post hoc analysis, missing data points and additional information are difficult to acquire. The initial study was completed with a relatively small number of patients receiving critical interventions, and treatment data demonstrate wide confidence intervals.

## Conclusions

Despite clear differences in the care delivered during emergency department resuscitation to patients with cardiogenic and non-cardiogenic shock types, we did not find any significant difference in actual care delivered between patients who were assessed using PoCUS and those who were not. This may help explain the previously reported lack of outcome differences between groups. The addition of PoCUS to usual clinical assessment is unlikely to impact outcomes unless it changes the care provided in a meaningful way.

## Appendices

Appendix 1: Clinicians’ Capsule

What is known about the topic?

Point-of-care ultrasound (PoCUS) is widely used in critically ill patients to guide treatment but has not been shown to improve mortality in undifferentiated hypotensive patients.

What did this study ask?

Did patients with various types of shock receive different care with and without PoCUS in a multicentre RCT?

What did this study find?

Although treatment differed by type of shock, there were no differences between patients who received PoCUS and those in the control group.

Why does this study matter to clinicians?

Clinicians’ treatment of cardiogenic and non-cardiogenic shock did not alter when PoCUS was added to usual care, a possible reason why no outcome differences were seen previously.
